# Syncope caused by congenital anomaly at the craniovertebral junction: a case report

**DOI:** 10.1186/1752-1947-8-330

**Published:** 2014-10-08

**Authors:** Naohisa Miyakoshi, Michio Hongo, Yuji Kasukawa, Yoichi Shimada

**Affiliations:** 1 Department of Orthopedic Surgery, Akita University Graduate School of Medicine, 1-1-1 Hondo, Akita 010-8543, Japan

**Keywords:** Craniovertebral junction, Syncope, Vertebral artery

## Abstract

**Introduction:**

Anomalies in the craniovertebral junction may be a rare cause of syncope. The mechanisms of syncope related to craniovertebral junction anomaly remain unknown.

We present an extremely rare case with anomaly in the craniovertebral junction and syncope, and discuss the mechanism of the syncope.

**Case presentation:**

A 10-year-old Japanese boy with a congenital anomaly in the craniovertebral junction presented with recurrent syncope. A physical examination showed generalized hyperreflexia, but motor and sensory examinations were normal. Computed tomography and magnetic resonance imaging showed basilar invagination and spinal cord compression at his craniovertebral junction. Three-dimensional computed tomography angiography revealed an anomalous course of his bilateral vertebral arteries, both of which showed a persistent first intersegmental artery that entered the spinal canal at the caudal portion of the C1 posterior arch. In this case, the arteries were nearly pinched between the C1 posterior arch and the pars interarticularis of the C2. C1 laminectomy and occiput-cervical fusion (O-C2) was performed using an instrumentation system. After surgery, the syncope was not observed.

**Conclusions:**

Syncope can be related to compression of extracranial arteries within the neck. In this case, transient brain ischemia caused by the anomalous course of vertebral arteries that were pinched between the C1 posterior arch and the pars interarticularis of C2 in cervical motion was the suspected cause of the syncope.

## Introduction

Syncope can arise through several mechanisms; one well-known cause is the vasodepressor reflex. Syncope can also be related to compression of the extracranial arteries in the neck [1]. Anomalies in the craniovertebral junction (CVJ) may be a cause of syncope, but appear to be extremely rare [2]. Mechanisms of syncope relating to CVJ anomalies remain unclear. We present here a pediatric case involving recurrent syncope and anomaly of the CVJ. The suspected mechanisms of syncope in the present case are discussed.

## Case presentation

A 10-year-old Japanese boy with a 2-year history of sleep attacks or syncope was referred to our orthopedic department because his mother noted that he had been experiencing frequent falls. He had been followed up by a pediatric orthopedist for asymptomatic congenital kyphoscoliosis with vertebral malformations in the thoracolumbar spine. He was treated for narcolepsy by a psychiatrist, but no clear diagnostic evidence was apparent, such as abnormalities on electroencephalography. On first presentation to this department, he suddenly lost consciousness. The attack seemed more like syncope than a narcoleptic episode. The mother said he experienced such attacks three to five times a day, with each episode lasting from 30 seconds to a few minutes. After each attack, he usually fell asleep. He showed neither motor weakness nor sensory loss in his upper or lower extremities, and no bladder dysfunction was evident, but generalized hyperreflexia was identified. Electrocardiography showed normal results, and we did not observe any bradycardia at the time of syncope. Breathing likewise remained normal during syncope.

**Figure 1:**
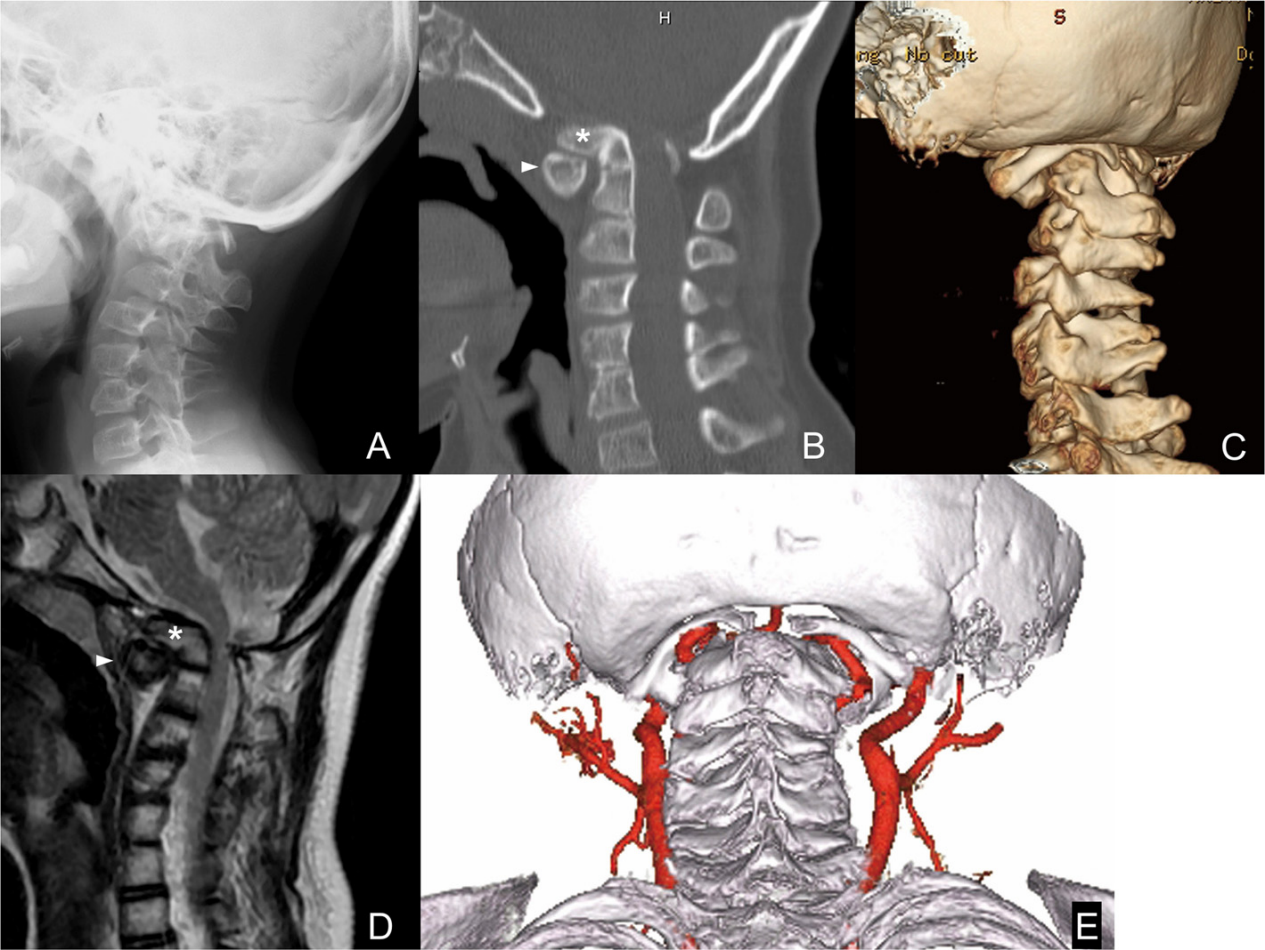
**Preoperative imaging of the craniovertebral junction. (A)** Lateral radiography shows malalignment of the craniovertebral junction. **(B)** Midsagittal reconstruction from computed tomography shows an anteriorly tilted odontoid process of C2 and migration of a hypoplastic C1 posterior arch into the foramen magnum. **(C)** Posterolateral view of three-dimensional computed tomography shows migration of the C1 posterior arch into the foramen magnum and rachischisis (spina bifida) of the C1 posterior arch. **(D)** Midsagittal T2-weighted magnetic resonance imaging shows compression of the spinal cord by the basilar invagination and an anteriorly shifted C1 posterior arch. **(E)** Posteroinferior view of three-dimensional computed tomography angiography shows anomalous courses of bilateral vertebral arteries (persistent first intersegmental artery), which were located between the C1 posterior arch and the pars interarticularis of C2. Asterisks, odontoid process; arrowheads, C1 anterior arch.

Plain lateral radiography of his CVJ showed abnormal alignment with a clivo-axial angle (the angle formed by lines drawn along the clivus and the posterior aspect of the odontoid process) of 112° (normal, 150 to 180°; Figure 1A) [3]. Lateral radiographies in flexion and extension positions showed significant instability between C1 and C2. Computed tomography (CT) and three-dimensional (3D)-CT of his CVJ showed an anteriorly tilted odontoid process of C2 and a hypoplastic C1 posterior arch with rachischisis (spina bifida) that was migrating into his foramen magnum (Figures 1B and 1C). His bilateral lateral atlantoaxial joints were dislocated anteriorly and his C1 anterior arch was hypertrophied. Magnetic resonance imaging showed basilar invagination and compression of his spinal cord at the C1 level (Figure 1D). Subsequent 3D-CT angiography showed abnormal courses of bilateral vertebral arteries (VAs; Figure 1E). Both VAs showed a “first persistent intersegmental artery” that entered his spinal canal at the caudal portion of the C1 posterior arch after emerging from the C2 transverse foramen, without passing through the C1 transverse foramen. These arteries appeared to be nearly pinched between the C1 posterior arch and the pars interarticularis of C2. The VAs at the subaxial spine were not hypoplastic. Based on these findings, we suspected that the cause of syncope was this arterial anomaly at his CVJ, and transient brain ischemia could be caused by these anomalous courses of the VAs being pinched between the C1 posterior arch and the pars interarticularis of C2 during cervical motions, especially in flexion. Posterior decompression and occiput-cervical fusion was therefore planned for decompression of the spinal cord and to prevent compression of the first persistent intersegmental arteries during motion.

**Figure 2:**
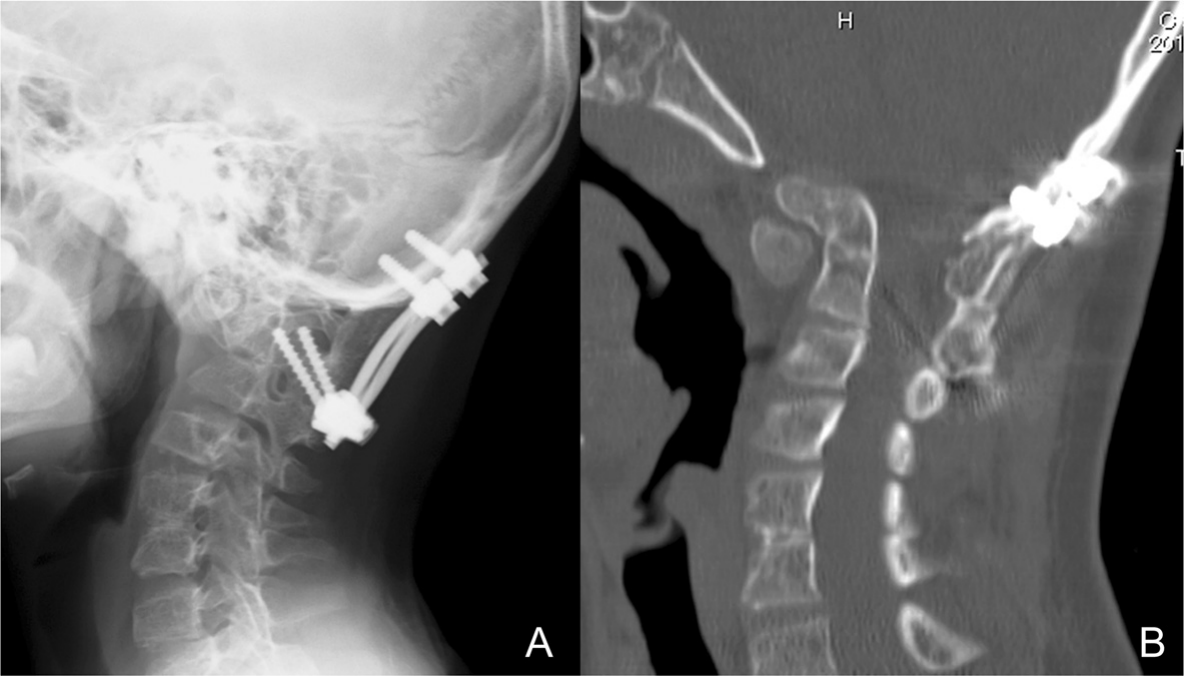
**Postoperative imaging of the craniovertebral junction. (A)** Lateral radiography shows improved alignment of the craniovertebral junction. **(B)** A midsagittal reconstruction of computed tomography obtained 4 months postoperatively shows complete bone union.

C1 laminectomy and posterior occiput-cervical fusion (O-C2) were performed using an instrumentation system (VERTEX® MAX; Medtronic Sofamor Danek, Memphis, TN, USA). Intraoperatively, the migrated C1 posterior arch was repositioned after skull traction and we were able to reach the upper surface of C1. Decompression of his foramen magnum was therefore not required for C1 laminectomy. A portion of the C1 posterior arch rachischisis consisted of tendon-like tissue that adhered severely to the dura. During C1 laminectomy, the locations of VAs were detected using Doppler ultrasonography. C2 pedicle screws were inserted with the assistance of a navigation system (Stealth Station®; Medtronic Sofamor Danek). After C1 laminectomy and screwing, monocortical autologous bone was harvested from the right posterior iliac crest and grafted between the decorticated posterior surfaces of the occipital bone and C2.The postoperative course was uneventful. Occiput-cervical alignment appeared improved after surgery, with a clivo-axial angle of 130° (Figure 2A). Since the surgery, he has not experienced any episodes of syncope. Cervical orthosis was applied for 6 weeks. Bone union was confirmed on CT at 4 months postoperatively (Figure 2B). As of the latest follow-up at 3.5 years postoperatively, he still showed no syncope, and was able to walk and run without falls.

## Discussion

Congenital anomalies in the CVJ can cause numerous symptoms [4]. In an analysis of 576 surgically treated patients with anomalies in the CVJ, motor dysfunction including weakness and spasticity, sensory dysfunction, dysfunction of the vestibular cerebellum including ataxia and nystagmus, cranial nerve dysfunction including swallowing difficulty and choking on water, and dyspnea were the most common clinical symptoms [4]. All these symptoms were neurological symptoms. Syncope may also occur in patients with CVJ malformation (Table 1), but appears to be extremely rare compared with the aforementioned symptoms.

**Table 1:** Reported cases of craniovertebral junction malformation and syncope

**Author**	**Age, sex**	**Suspected cause of syncope**	**Symptoms other than syncope**
Corbett *et al.*[2]	29 M	Intolerance of Valsalva-induced changes relating to CVJ malalignment and Chiari malformation	Dizziness
Pratiparnawatr *et al.*[5]	31 M	Unclear	Nystagmus, fasciculation of tongue, atrophy of tongue and sternocleidomastoid muscle, weakness of extremities
Present	10 M	Bilateral persistent first intersegmental arteries pinched between the C1 posterior arch and the pars interarticularis of C2	Frequent falls

CVJ, craniovertebral junction; M, male.

Corbett *et al*. [2] reported a case of syncope precipitated by sneezing in a 29-year-old man in association with Arnold–Chiari type I malformation and basilar invagination. In that case, suboccipital craniectomy and C1-C2 laminectomies alleviated the symptoms and the authors speculated that an abnormally acute clivo-axial angle (120°), small foramen magnum, and type I Chiari malformation appeared to represent a combination of features intolerant of Valsalva-induced changes in cerebral volume, brainstem position, cerebrospinal fluid dynamics, or blood vessel relationships [2]. Pratiparnawatr *et al*. [5] reported the case of a 31-year-old man with basilar invagination and syringomyelia who showed cough-induced syncope. However, that patient also exhibited downbeat nystagmus, atrophy and fasciculation of the right side of the tongue, atrophy of the right sternocleidomastoid muscle, mild weakness of the extremities, and generalized hyperreflexia. The cause of syncope was unclear in that case [5].

In the present case, although neurological factors might also have been involved, we speculated that brain ischemia caused by direct compression of bilateral anomalous VAs relating to the CVJ malformation was the major cause of syncope. Two types of anomalous course of VAs have been reported [6-9]. One is a “fenestration”, showing VA duplication after emerging from the C2 transverse foramen; one branch enters the spinal canal between C1 and C2 and the other runs normally, passing through the C1 transverse foramen and entering the spinal canal on the cranial side of the posterior arch. The other is a “persistent first intersegmental artery (without persistence of the primary VA)”, showing the artery entering the spinal canal at the caudal portion of the C1 posterior arch after emerging from the C2 transverse foramen, without passing through the C1 transverse foramen. Incidences of these two VA anomalies have been reported as 0.24 to 1.0% and 0.6 to 4.7%, respectively, in 300 to 1436 patients who had no symptoms from cervical spine lesions [6-8]. Usually, these VA anomalies occur unilaterally [6,9,10], and bilateral cases appear to be rare. Hong *et al*. [6] reported that the incidence of bilateral persistent first intersegmental artery among 1013 subjects was 0.8% compared with 3.8% for unilateral cases.

Our patient showed bilateral persistent first intersegmental arteries. Bilateral persistent first intersegmental arteries usually exist on the surface of the soft dura at the C1 level, so spinal motion does not interfere with blood flow in the anomalous artery. However, in the present case, 3D-CT angiography clearly showed the arteries lying between the two hard structures of the C1 posterior arch and the pars interarticularis of C2. The persistent first intersegmental arteries in this patient must therefore have been pinched at this level by the C1 posterior arch and pars interarticularis of C2 during cervical motions, particularly in flexion. Subsequent changes to blood flow in the basilar artery would then have induced transient cerebral ischemia, resulting in syncope. In addition, the fact that this patient only experienced minor neurological symptoms (hyperreflexia and frequent falls, but no motor or sensory deficits) supports the hypothesis that transient brain ischemia caused by the anomalous courses of the VAs was the major cause of syncope in this case. Bow hunter’s syndrome, also referred to as rotational VA occlusion syndrome [11], is a rare vascular phenomenon that can be a differential diagnosis for transient cerebral ischemia caused by VA compression. However, bow hunter’s syndrome usually occurs with cervical rotation in patients with an anatomically normal skeleton and VAs, whereas syncope in the present case was not induced by cervical rotation but rather suspected flexion in a patient with skeletal anomaly and bilateral anomalous VAs. We therefore excluded the possibility of bow hunter’s syndrome in the present case.

## Conclusions

We have presented an extremely rare case of congenital anomaly in the CVJ resulting in recurrent syncope. Posterior decompression and occiput-cervical fusion relieved all symptoms. Symmetrical anomaly of the VAs (bilateral persistent first intersegmental arteries) pinched by the osseous structures (C1 posterior arch and pars interarticularis of C2) during occipital-cervical motion was the suspected cause of syncope.

## Consent

Written informed consent was obtained from the patient’s legal guardians for publication of this case report and the accompanying images. A copy of the written consent is available for review by the Editor-in-Chief of this journal.

## Abbreviations

3D: Three-dimensional; CT: Computed tomography; CVJ: Craniovertebral junction; VA: Vertebral artery.

## Competing interests

The authors declare that they have no competing interests.

## Authors’ contributions

NM performed the surgery and prepared the manuscript. MH, YK, and YS assisted in drafting the manuscript and reviewed the article. All authors read and approved the final manuscript.
